# Quantification of the Geranium Essential Oil, Palmarosa Essential Oil and Phenylethyl Alcohol in *Rosa damascena* Essential Oil Using ATR-FTIR Spectroscopy Combined with Chemometrics

**DOI:** 10.3390/foods10081848

**Published:** 2021-08-11

**Authors:** Nur Cebi

**Affiliations:** Department of Food Engineering, Faculty of Chemical and Metallurgical Engineering, Yıldız Technical University, İstanbul 34210, Turkey; nurcebi@yildiz.edu.tr

**Keywords:** FTIR, *Rosa damascena* essential oil, PLSR, PCR, HCA, PCA, adulteration, geranium essential oil, palmarosa essential oil, phenyl ethyl alcohol

## Abstract

*Rosa damascena* essential oil is an essential oil that has the greatest industrial importance due to its unique quality properties. The study used ATR-FTIR (attenuated total reflectance-Fourier transform infrared) spectroscopy coupled with chemometrics of PLSR (partial least squares regression) and PCR (principal component regression) for quantification of probable adulterants of geranium essential oil (GEO), palmarosa essential oil (PEO) and phenyl ethyl alcohol (PEOH). Hierarchical cluster analysis was performed to observe the classification pattern of *Rosa damascena* essential oil, spiked samples and adulterants. *Rosa damascena* essential oil was spiked with each adulterant at concentrations of 0–100% (*v*/*v*). Excellent R^2^ (regression coefficient) values (≥0.96) were obtained in all PLSR and PCR cross-validation models. The SECV (standard error of cross-validation) values ranged between 0.43 and 4.15. The lowest SECV and bias values were observed in the PLSR and PCR models, which were built by using the raw FTIR spectra of all samples. Hierarchical cluster analysis through Ward’s algorithm and Euclidian distance had high potential to observe the classification pattern of all adulterated and authentic samples. In conclusion, the combination of ATR-FTIR spectroscopy with multivariate analysis can be used for rapid, cost-effective, easy, reliable and high-throughput detection of GEO, PEO and PEOH in *Rosa damascena* essential oil.

## 1. Introduction

*Rosa damascena* (Damask rose) is one of the most important species of the genus Rosa, which consists of at least 200 species. It is known as a unique type of oil-bearing rose with its intense and pungent scent [1]. *Rosa damascena* is mainly cultivated in Turkey and Bulgaria; rose oil, concrete and absolute are the major products obtained from *Rosa damascena* [2]. Turkey is the main rose oil producer in the world and provides approximately 50% of the supply [1].

*Rosa damascena* essential oil is defined as: essential oil obtained by steam distillation of the flowers of *Rosa damascena* Miller of the Rosaceae family, cultivated in Turkey, Morocco and Bulgaria according to the international standard [3]. The blooming of *Rosa damascena* starts in early May and continues to the beginning of July, in Isparta (Turkey). In the flowering season, the roses are picked in the very early hours of the morning and then they are transferred to the distillation facilities [4]. Previous studies reported that 3500–4000 kg of rose flowers are needed to produce 1 kg of rose essential oil by the distillation process in the industrial facilities [5].

*Rosa damascena* essential oil is a type of rose oil that has the highest economic value for the perfume, cosmetics, pharmaceutical and food industries with its sharp and intense scent making it distinctive among other scented rose types [1]. It is widely included in cosmetic products such as creams, shampoos, soaps, lotions and more, and is also used in foodstuffs such as puddings, jellies, candies, delights and chocolate [6]. Previous studies reported that *Rosa damascena* is known as fluid gold due its high price and economic importance [7].

The chemical composition of *Rosa damascena* includes monoterpene alcohols (e.g., citronellol, geraniol, nerol), the pyran class of monoterpenes (e.g., rose oxide), metabolites related with the shikimic pathway (e.g., methyl eugenol and phenyl ethyl alcohol), long-chain hydrocarbons (nonadecane, nonadecene, eicosane and heneicosane), and metabolites resulting from carotenoids’ degradation (damascenones and β-ionones) [8]. A number of studies reported that high-value natural product *Rosa damascena* essential oil has antimicrobial, antioxidant, relaxant, anti-inflammatory and insecticidal properties due to the biologically active compounds such as citronellol, geraniol, nerol, linalool and phenyl ethyl alcohol in its composition [8].

The high production costs, limited production quantity and wide industrial applications of *Rosa damascena* essential oil are the biggest pressures driving economically motivated adulteration. This essential oil is one of the most expensive in the essential oil market and this situation makes it highly prone to adulteration with cheaper oils or synthetic materials. Adulteration of natural products such as essential oils may cause dishonest trading, exploitation of consumers, deterioration of the authentic product, food-safety related problems and health risks.

Previous studies reported that adulteration of *Rosa damascena* essential oil (REO) is implemented by blending it with geranium oil (GEO), palmarosa oil (PEO) and phenyl ethyl alcohol (PEOH) [9]. Geranium essential oil is mainly composed of citronellol (30.2%), citronellyl formate (9.3%) and geraniol (7.6%) [10]. Additionally, major volatile compounds of the palmarosa essential oil are linalool (2.6–3.8%), geraniol (91.8–92.8%) and geranial (1.8–2.0%) [11].

There is a need for new methodologies for the detection and quantification of such adulterants in its composition. The FTIR (Fourier transform infrared) spectroscopy technique provides fast, efficient, trustable, easy, eco-friendly and economical determination of fingerprinting data related to the overall chemistry of the material investigated [12]. Such FTIR analyses could be performed using either no or only minimum sample preparation with very small amounts of essential oil. It is now well-established from a variety of studies that essential oil quality can be successfully evaluated using Fourier transform infrared spectroscopy combined with chemometric techniques [13]. Previous studies reported that vibrational spectroscopy combined with chemometric models were effectively used for the determination of quality in lavender oils [14]. Similarly, Raman and ATR-FTIR (attenuated total reflectance-Fourier transform infrared) spectroscopy techniques were successfully applied for quality evaluation of eucalyptus essential oil [15]. Sandasi et al. (2011) presented that vibrational spectroscopy and chemometrics can be used for the quality assessment of rose-scented geranium [16]. Some recent studies showed that various adulterants could be successfully quantified in lemon essential oil and mentha piperita essential oil by using FTIR spectroscopy combined with chemometrics [12,17].

To the best of our knowledge, the current research is the first attempt at the detection and quantification of PEO, GEO and PEOH in *Rosa damascena* essential oil by using FTIR spectroscopy combined with the chemometrics of partial least squares regression (PLSR) and principal component regression (PCR). Additionally, hierarchical cluster analysis (HCA) and principal component analysis (PCA) was applied for visualization of the cluster pattern of adulterants, adulterated samples and *Rosa damascena* essential oil.

## 2. Materials and Methods

### 2.1. Apparatus

A Bruker Tensor 27 FTIR spectrometer equipped with an attenuated total reflectance (ATR) accessory was used for spectral acquisition at the spectral range of 4000–650 cm^−1^. The FTIR spectrometer had a KBr beamsplitter and DLaTGS detector. The attenuated total reflectance (ATR) unit was used in combination with the FTIR spectrometer. Spectral acquisition and instrument control was assured by OPUS Version 7.2 (Bruker, Karlsruhe, Germany).

### 2.2. Essential Oils and Spiked Samples

Original *Rosa damascena* essential oils (REO1, REO2, REO3), geranium essential oils (GEO1, GEO2, GEO3) and palmarosa essential oils (PEO1, PEO2, PEO3) were purchased from well-known companies (Istanbul, Turkey). Phenyl ethyl alcohol (PEOH) was purchased from Sigma-Aldrich (Darmstadt, Germany). Spiked (adulterated) samples were diligently prepared at the adulteration levels of 0, 2%, 4%, 8%, 16%, 32%, 64% and 100% *v*/*v* for each adulterant. Forty-two spiked samples were prepared in total. Cross-validation graphs were drawn using eight levels of concentration. All of the samples were stored at 4 °C in dark vials until the FTIR measurements.

### 2.3. ATR-FTIR Measurements

The FTIR spectra of all samples (authentic essential oils, spiked samples and adulterants) were measured at the spectral range of 4000–600 cm^−1^. Twenty (20) µL of each sample was dripped on the ATR crystal with the help of an automatic pipette (20–200 µL). The ATR-FTIR spectra of all samples were recorded with a resolution of 4 cm^−1^, accumulating 16 scans per spectra. The spectral acquisition was performed against the background air spectrum. Each sample was measured three times and an average spectrum was obtained. After data acquisition, the diamond crystal was cleaned using ethyl alcohol (98%) and then dried with soft tissues.

### 2.4. Chemometrics

#### 2.4.1. Classification by Hierarchical Cluster Analysis (HCA) and Principal Component Analysis (PCA)

Classification of *Rosa damascena* essential oils, spiked samples and adulterants (PEO, GEO and PEOH) was accomplished by using the HCA function of OPUS (Version 7.2) (Bruker, Germany software of the FTIR equipment. Additionally, principal component analysis was performed by using the PCA function of OPUS (Version 7.2) software of the FTIR equipment. In the HCA, the classification pattern of all the samples was obtained using the second derivative and vector normalized FTIR spectra at the spectral range of 3541–3153 cm^−1^ and 1771–663 cm^−1^.

#### 2.4.2. Construction of PLSR and PCR Calibration Models

Multivariate data analysis based on partial least squares regression (PLSR) and principal component regression (PCR) was implemented using the GRAMS 32 software (Galactic Industries Corp., Salem, NH, USA). Calculation of the quantities of adulterants palmarosa essential oil, geranium essential oil and phenyl ethyl alcohol was accomplished using the raw, first derivative and second derivative FTIR spectra of all samples. The quantification levels were constructed as 0%, 2%, 4%, 8%, 16%, 32%, 64% and 100% (*v*/*v*) for each adulterant. The mid infrared (MIR) spectra of adulterated *Rosa damascena* essential oil samples with three different adulterants were collected. An optimum number of factors were selected for calibration and cross-validation on the basis of the PRESS (residual sum of squares) value from the regression models. The accuracy of the models was determined and compared on the basis of the standard error of cross-validation (SECV) value. The different spectral range in which concentration alteration was clearly monitored was selected for each adulterant to build regression models.

## 3. Results

### 3.1. ATR-FTIR Spectra of Rosa Damascena Essential Oil and Adulterants

**Figure 1:**
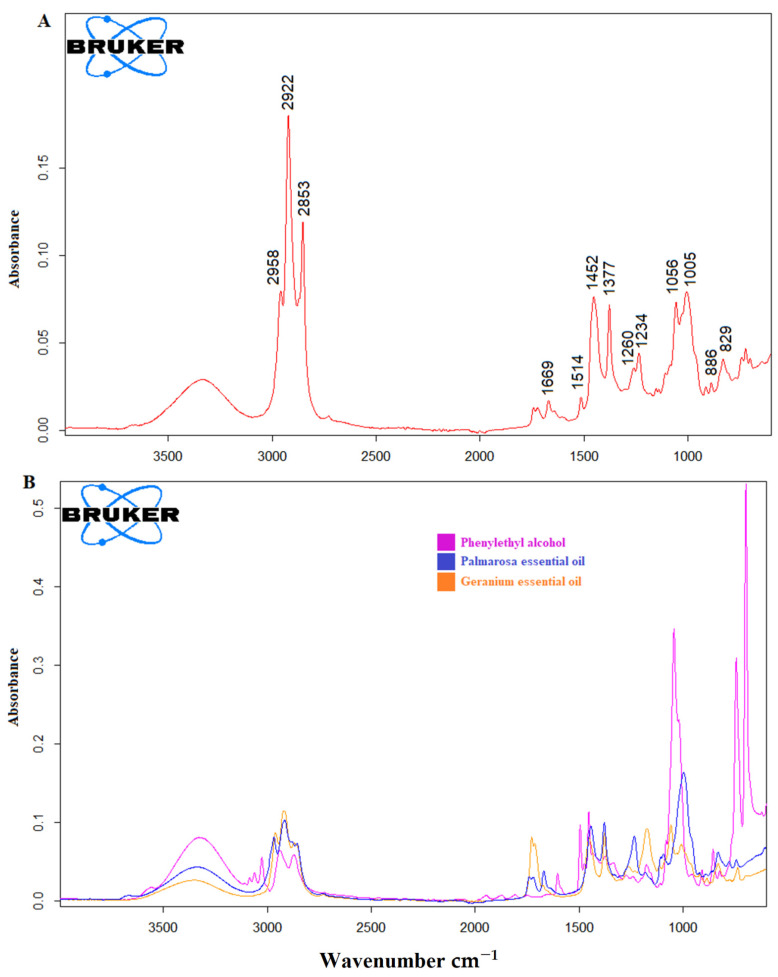
Fingerprint ATR-FTIR spectrum of *Rosa damascena* essential oil (REO) at the spectral region of 4000–600 cm^−1^ (**A**) ATR-FTIR spectra of palmarosa essential oil (PEO), geranium essential oil (GEO) and phenyl ethyl alcohol (PEOH) (**B**).

The main principle of infrared spectroscopy is built on the interactions of chemical bonds of a sample by the radiation of an infrared light source to produce a signature fingerprint in the form of a spectrum [18]. In other words, an IR spectrum of a material is specific and unique for it and presents the chemical information in the form of a two-dimensional spectrum. The ATR-FTIR spectra of *Rosa damascena* essential oil and the adulterants (GEO, PEO and PEOH) are presented in Figure 1A,B, respectively. The FTIR spectrum of the *Rosa damascena* essential oil was quite similar to the one obtained in previous study [1]. However slight differences were observed in the wavelengths of the bands due to the brand differences. The bands at 2922 and 2853 cm^−1^ were due to the methylene C–H asymmetric and symmetric stretching vibrations, respectively [1]. The peak at 1669 cm^−1^ may be attributed to the C=C stretching vibrations [19]. The band at 1514 cm^−1^ may be due to the aromatic ring C=C skeleton vibrations [20] Two bands at 1452 cm^−1^ and 1377 cm^−1^ were attributed to the C- H bending vibrations and C–H asymmetric + symmetric bending vibrations, respectively [1,21]. Two bands at 1260 and 1235 cm^−1^ can be assigned to the C–C–O stretching vibrations and C–O stretching vibrations of phenolics [22]. The bands at 1056 cm^−1^ and 1005 cm^−1^ can be due to the OH group vibrations and C-H bending vibrations, respectively [1,21]. The ATR-FTIR spectra of palmarosa essential oil, geranium essential oil and phenyl ethyl alcohol are presented in Figure 1B. The GEO, PEO and PEOH were marked with orange, blue and pink colors, respectively. As can be seen, each of the adulterants showed distinct spectral properties. Observed bands and their assignments are presented in Table 1.

### 3.2. Interpretation of the Hierarchical Cluster Analysis (HCA) and Principal Component Analysis (PCA) Results

Hierarchical cluster analysis was applied to monitor the classification pattern of all the evaluated samples on the basis of their resemblance and diversities. Previous studies reported that hierarchical cluster analysis showed high capability for easy, rapid and accurate chemotaxonomy characterization [23]. In the previous studies, HCA was applied to the gas chromatography-flame ionization detection (GC-FID) results of *Rosa damascena* absolute samples through the Euclidian distance. Their results showed that HCA was successfully applied for the classification of rose absolute samples for authenticity control [24]. Another study presented that HCA was effectively applied for the classification of olive oil samples on the basis of FTIR spectral data and similarities and dissimilarities were clearly observed [25].

**Figure 2:**
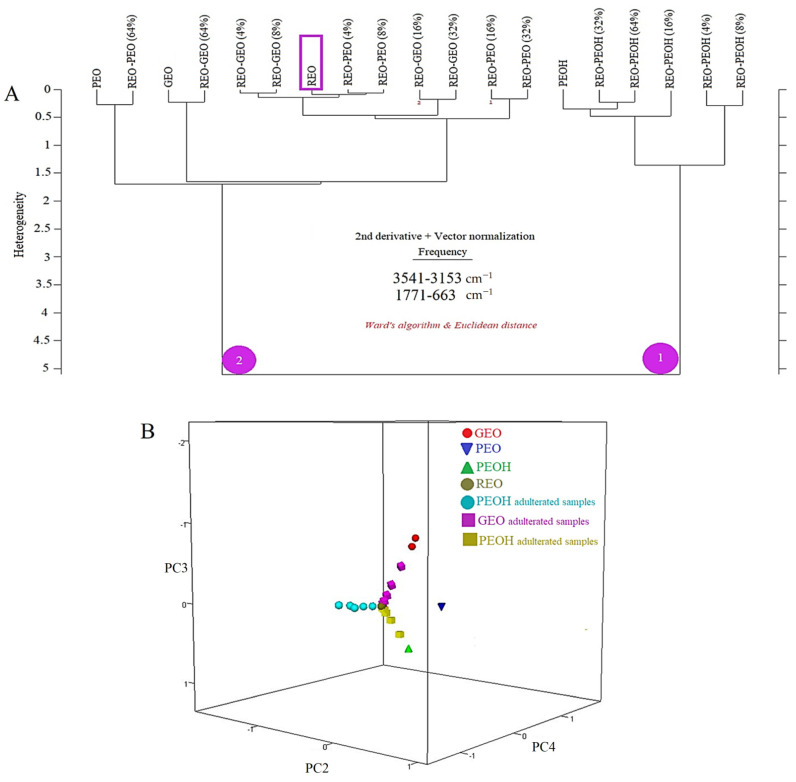
Two-dimensional HCA dendrogram of the *Rosa damascena* essential oil, adulterated samples, palmarosa essential oil, geranium essential oil and phenyl ethyl alcohol (Ward’s algorithm, Euclidian distance) (**A**). Three dimensional PCA plot of the *Rosa damascena* essential oil, adulterated samples, palmarosa essential oil, geranium essential oil and phenyl ethyl alcohol (**B**).

In the current research, HCA was applied to the *Rosa damascena* essential oil (REO), palmarosa essential oil (PEO), geranium essential oil (GEO) and adulterated samples. The HCA was performed using the second derivative and vector normalized FTIR spectra of all samples in the spectral range of 3541–3153 cm^−1^ and 1771–663 cm^−1^. The 2-D HCA graph is presented in Figure 2A. The HCA dendrogram displays the interrelationships between the elements investigated. Two main branches were observed on the HCA dendrogram. Phenyl ethyl alcohol (PEOH) and PEOH adulterated samples were clustered on the right arm (numbered as 1) of the dendrogram. As can be seen, PEOH adulterated samples showed a classification pattern related to the spiking concentration. The low PEOH spiking concentrations (4% and 8%) were clustered on the right side of the dendrogram. High PEOH spiking concentrations (16%, 32% and 64%) were clustered near to the 100% PEOH. These results indicate that PEOH and PEOH-adulterated samples were distinctively classified from GEO and PEO adulterated samples. What is interesting about the dendrogram in Figure 2A is that it not only monitored the clustering of all the samples, but also provided spiking level-related classification patterns of all samples. On the left arm of the dendrogram (numbered as 2), PEO, GEO and the highest spiking level of 64% were distinctively classified apart from other PEO and GEO adulterated samples. The *Rosa damascena* essential oil (REO) is located very close to the PEO adulterated (4%) samples. The nearest samples to the REO essential oil were GEO and PEO adulterated samples at the spiking levels of 4% and 8%. Overall, these results indicate that hierarchical cluster analysis through Ward’s algorithm and Euclidian distance had high capability to reveal PEO and GEO adulteration in *Rosa damascena* essential oil. Additionally, 3-D PCA plot is presented in Figure 2B, classification pattern of REO, GEO, PEO, PEOH and adulterated samples are observed in 3-D PCA plot. REO samples were clustered at the center of the plot near to the adulterated samples. PEO, GEO and PEOH samples were clustered distinctly from REO and adulterated samples. As result, when we compare HCA and PCA, it is seen that HCA dendrogram provided a comprehensive understanding of classification pattern of all samples.

### 3.3. Prediction of GEO, PEO and PEOH Contents of Rosa Damascena Essential Oil Using PLSR and PCR Calibration Models

Partial least squares regression (PLSR) and principal component regression (PCR) calibration models that have the capability to extract specific information from big data sets are widely used for quantification of compounds investigated in various food matrices such as juices, teas, milk powders, alcoholic beverages and margarine using FTIR data sets [26]. Additionally, recent studies showed that PLSR regression models have been used for the quantification of specific bioactive compounds in essential oils [27]. In general, PLSR and PCR models are used to build regression models that construct a linear relationship between the concentration and intensity of an analyte. The PLSR extracts variables called latent variable or PLS-factor that explain most of the variance from the spectra (X) and concentration (Y) and minimizes the irrelevant variations in the X matrix [28]. Both PCR and PLSR are based on data compression and inverse calibration models and it is possible to build calibration models for the desired or selected compounds [29].

**Figure 3:**
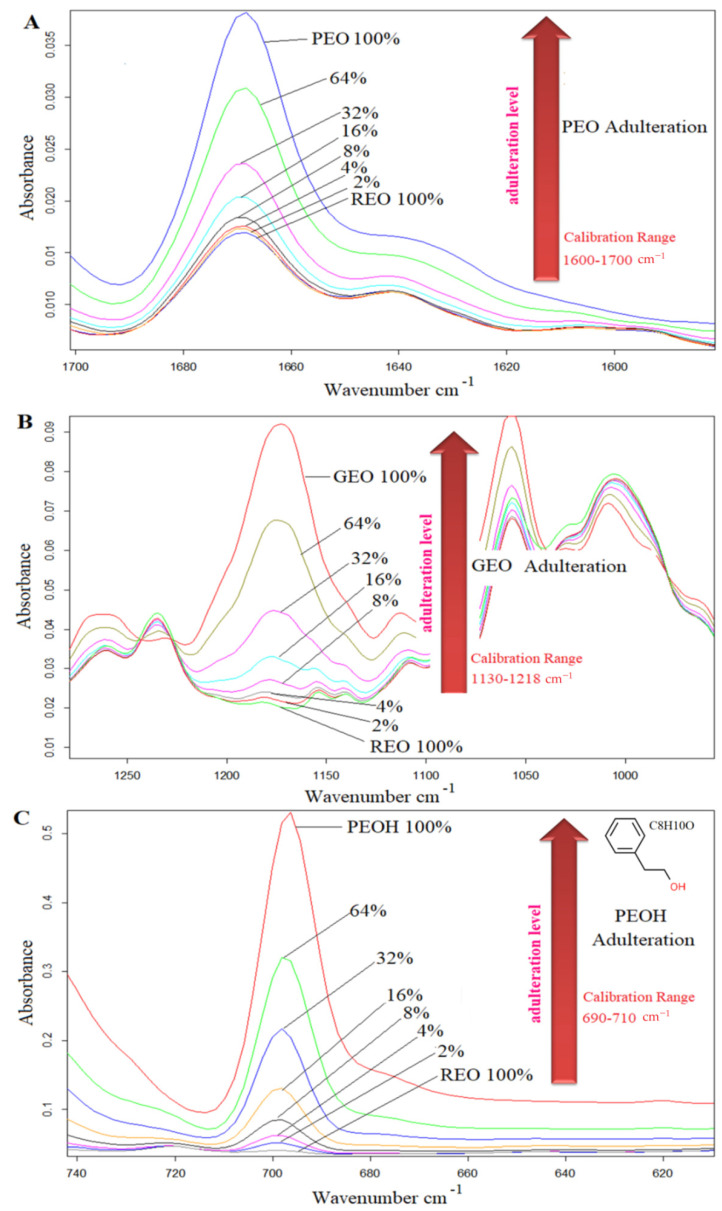
Concentration-related selected spectral ranges, (**A**) Palmarosa essential oil adulteration in *Rosa damascena* essential oil, (**B**) Geranium essential oil adulteration in *Rosa damascena* essential oil (**C**) Phenylethyl alcohol adulteration in essential oil adulteration in *Rosa damascena* essential oil.

**Figure 4:**
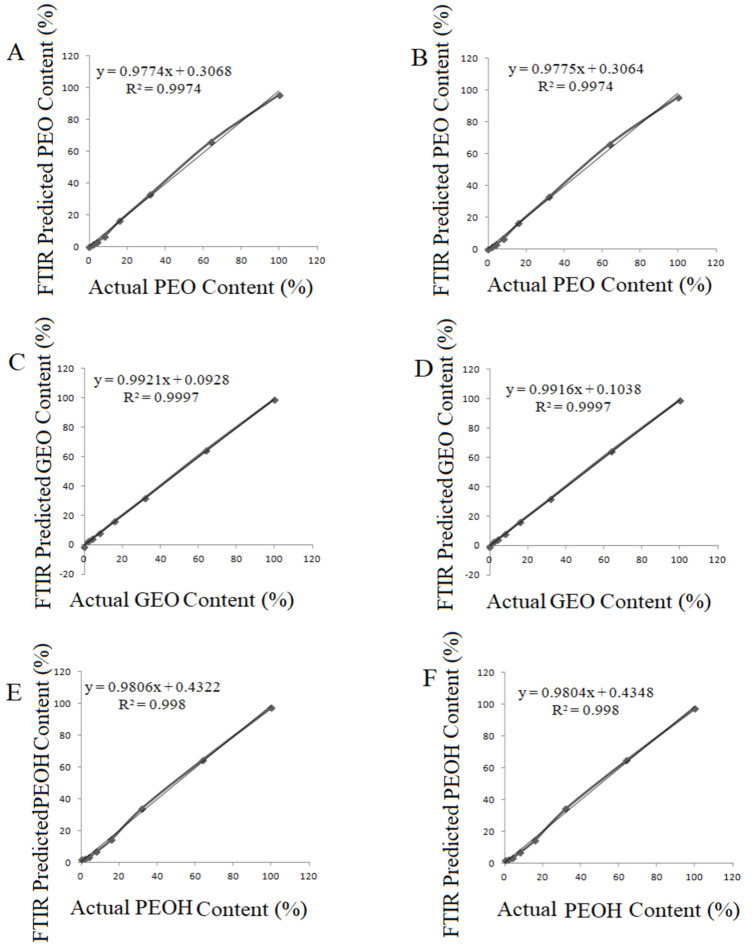
Cross-validation plot (PLSR-normal spectra) PEO adulterated REO (**A**); Cross-validation plot (PCR-raw spectra) PEO adulterated REO (**B**); Cross-validation plot (PLSR-raw spectra) GEO adulterated REO (**C**); Cross-validation plot (PCR-raw spectra) GEO adulterated REO (**D**); Cross-validation plot (PLSR-raw spectra) PEOH adulterated REO (**E**); Cross-validation plot (PCR-raw spectra) PEOH adulterated REO (**F**).

Previous studies reported that PLS and PCR techniques were successfully applied to the FTIR data of various oils [30]. In the present study, PLSR and PCR calibration models were built for the prediction of the quantity of adulterants (PEO, GEO and PEOH) in *Rosa damascena* essential oil. Quantification of spiked adulterants was performed using the selected spectral ranges for each adulterant. Selection of the spectral regions was implemented on the basis of the concentration-related alterations in the spectral ranges. The spectral region in which the concentration change of the analyte could be observed was selected as the calibration range for quantification. Selected spectral ranges and concentration changes of PEO, GEO and PEOH are presented in Figure 3A–C, respectively. The spectral ranges of 1700–1600 cm^−1^, 1218–1130 cm^−1^ and 710–690 cm^−1^ were selected for PEO, GEO and PEOH, respectively. One can clearly see that the intensity of the band for each adulterant significantly increased with rising adulteration levels from 0% (100% REO) to 64% (Figure 3). Cross-validation curves of PLSR and PCR analyses are presented in Figure 4 for raw spectra.

Calibration levels were determined as 0%, 2%, 4%, 8%, 16%, 32%, 64% and 100% (*v*/*v*) for each adulterant. The term actual concentration represents the spiked adulterant concentrations and the term FTIR predicted concentration refers to the computed concentration of adulterant on the basis of the FTIR spectra [31].

The cross-validation results of PLSR and PCR models, calibration parameters, correlation coefficient (R^2^), standard error of cross-validation (SECV) and PRESS values are presented in Table 2. The benefit of cross-validation is better outlier detection. Cross-validation is the only validation method that can give complete outlier detection for the training set data. Since each sample is left out of the models during the cross-validation process, it is possible to calculate how well the spectrum matches the model by calculating the spectral reconstruction. Cross-validation parameters of regression model, preprocessing, the optimum number of factors, equation, regression coefficient, PRESS and SECV are presented in Table 2.

The accuracy of the regression models was evaluated on the basis of the regression coefficient (R^2^), SECV and bias values. The R^2^ value is a statistical measure in a regression model that determines the proportion of variance in the dependent variable that can be explained by the independent variable. The R^2^ value gives information about how favorable data fit the regression model. All of the models (PLSR and PCR) fit the adulterant (PEO, GEO and PEOH) concentrations well, with R^2^ values between 0.9850 and 0.9998. In other words, the regression models performed excellently to predict adulterant concentration in the *Rosa damascena* essential oil on the basis of the FTIR data. Previous studies reported that calibration models having an R^2^ value above 0.91 are considered to be excellent [32]. Additionally, the R^2^ value (≥0.96) of the plots between predicted and actual values were reported to be excellent in previous contributions [33].

Bias is defined as the systematic error between predicted and reference values, and bias value is computed as an average value of residuals [34]. Bias value gives information about the predictive ability of the model [35]. In the present study, the bias values ranged between 0.28 and 3.57. In general, favorable bias values were obtained. Previous researches reported that the SECV is the best single estimate of the prediction capability of the equation and the highest R^2^ value is chosen to develop calibration models [36]. In other words, the model that has the lowest SECV values and the highest R^2^ value has the most favorable capability to fit the linear relationship between actual adulterant contents and predicted adulterant contents by FTIR spectroscopy. In the current research, the SECV values ranged between 0.43 and 4.15. The lowest SECV and bias values were observed in the PLSR and PCR models which were built using the raw FTIR spectra of all samples. Raw (unprocessed) FTIR spectra resulted in better PLSR and PCR models when compared to the processed (first derivative and second derivative) ones. (Table 2).

Results from the current research showed that palmarosa essential oil (PEO), geranium essential oil (GEO) and phenyl ethyl alcohol (PEOH) adulteration in the *Rosa damascena* essential oil could be quantified successfully using the PLSR and PCR models at the adulteration range of 0–100% (*v*/*v*).

## 4. Discussion

Previous publications reported that FTIR spectroscopy combined with chemometrics of PLSR and PCR were effectively used for rapid, easy, economical, reliable and eco-friendly quantification of adulterants in various complex food matrices [26]. Unfortunately, as previously reported, “Unscrupulous producers have begun to fraudulently increase profits while keeping down raw material costs mainly through the addition of cheaper oils or oil constituents” [37]. The *Rosa damascena* essential oil is one of the most unique and high-cost essential oils with tremendous economic importance. Quite limited studies have been performed for evaluation of the authenticity and adulteration of *Rosa damascena* essential oil. Pellati et al. (2012) utilized gas chromatography combined with mass spectrometry, flame ionization detection and elemental analyzer/isotope ratio mass spectrometry for the determination of the authenticity of *Rosa damascena* Mill [38]. Previous studies reported that ultra-high mass spectrometry (UHPLC/TOFMS) was used for the determination of botanical and geographical origins of *Rosa damascena* and detected adulterations. Krupcik et al. (2015) applied enantioselective analysis of Bulgarian and Turkish *Rosa damascena* Miller essential oils for determination of their authenticity [39]. Cebi et al. (2021) evaluated the effectiveness of Fourier transform infrared spectroscopy, Raman spectroscopy and gas-chromatography mass spectrometry combined with chemometrics for the determination of the authenticity of *Rosa damascena* essential oil. A different study used liquid-liquid microextraction-gas chromatography mass spectroscopy for evaluation of adulteration in *Rosa damascena* essential oil [40]. All of these studies were important contributions to the scientific knowledge. However, none of them specifically investigated the highly probable adulterants of palmarosa essential oil, geranium essential oil and phenyl ethyl alcohol adulteration in *Rosa damascena* essential oil.

To the best of our knowledge, this research provides the first scientific findings about development of cross-validation models (PLSR and PCR) for calculation of the PEO, GEO and PEOH adulterant contents in *Rosa damascena* essential oil. The results showed that tracking of adulterants could be accomplished using analyte-related spectral regions in the mid-infrared spectral region. Quantification of each adulterant was accomplished with favorable R^2^, SECV and bias values. Previous contributions showed that FTIR spectroscopy coupled with regression models of PLSR and PCR had high capability of quantifying adulterants in essential oils [13,17]. The effectiveness of FTIR spectroscopy arises from its fingerprinting properties. An IR spectrum of material presents an image of overall chemical composition that can be utilized to build a robust taxonomic classification [41]. Chemometrics methods, such as PLSR and PCR, are used to find hidden information in the multivariate data from instruments such as FTIR In other words, the combination of infrared spectroscopy and multivariate calibration techniques provides an opportunity for quantification of adulterants on the basis of marker data [26].

## 5. Conclusions

This research study showed that FTIR spectroscopy coupled with multivariate analysis of PLSR and PCR could be used for quantification of palmarosa essential oil and geranium essential oil and phenylethyl alcohol in the *Rosa damascena* essential oil. We have shown that it is possible to discriminate between authentic *Rosa damascena* essential oil and adulterated oils. Hierarchical cluster analysis was applied to monitor the classification pattern of adulterated and pure essential oil samples. PLSR and PCR were performed for the prediction of adulterant contents at the concentration range of 0–100%. Excellent R^2^ values (≥0.96) were obtained in all cross-validation models of PLSR and PCR for normal, first derivative and second derivative FTIR spectra. The lowest SECV and bias values were obtained in PLSR and PCR models of raw spectra. In keeping with the original goals, the methodologies developed use existing ATR-FTIR equipment, do not require toxic or harmful chemicals, require minimum or no sample preparation, can be performed rapidly in a few minutes and do not need intensive labor or incur high application costs.

Applied scientific knowledge and techniques are adaptable to new and challenging adulteration and authenticity issues. In future studies, the development of new analytical methodologies coupled with chemometrics will help with the quality control of natural products such as high-cost essential oils in government controlled laboratories. Additionally, integration of well-built methodologies into hand-held infrared spectrometers will help to ensure quality control of essential oils in the field.

## Figures and Tables

**Table 1:** FTIR bands observed in spectra from *Rosa damascena* essential oil [1,18,19,20,21,22].

Band (cm^−1^)	Band Assignment
3345	O–H stretching
2958	methyl C–H stretching
2922	C–H asymmetric stretching
2853	C–H symmetric stretching
1669	C=C stretching
1514	C=C stretching
1452	C–H bending
1377	C–H bending
1260	C–C–O stretching
1234	C–O stretching
1056	O–H stretching
1005	C–H bending
829	C–H stretching

**Table 2:** PLSR (partial least squares regression) and PCR (principal component regression) calibration parameters and cross-validation results.

Sample Codes	Model	Factors	Preprocessing	Calibration (Cross-Validation)		Press	SECV	Bias
				Equation	Regression Coefficient			
PEO1 REO1 (PEO1 adulterated REO1)	PLSR	3	Raw	y = 0.9598x + 0.6122	R² = 0.9969	34.57	2.08	1.96
		3	First derivative	y = 0.9549x + 0.6494	R² = 0.9913	77.92	3.12	2.78
		3	Second derivative	y = 0.9528x + 0.7053	R² =0.9922	107.36	3.66	2.64
	PCR	3	Raw	y = 0.9669x + 0.4623	R² =0.9935	52.06	2.55	2.31
		3	First derivative	y = 0.955x + 0.6493	R² = 0.9913	86.83	3.29	2.78
		3	Second derivative	y = 0.9528x + 0.7065	R² = 0.9923	101.95	3.57	2.64
PEO2 REO2 (PEO*2* adulterated REO2)	PLSR	3	Raw	y = 0.9774x + 0.3068	R² = 0.9974	52.08	2.55	1.50
		3	First derivative	y = 0.9427x + 0.8437	R² = 0.9937	80.15	3.16	2.75
		3	Second derivative	y = 0.9405x + 0.9284	R² = 0.9921	61.41	2.77	2.91
	PCR	3	Raw	y = 0.9775x + 0.3064	R² = 0.9974	36.78	2.14	1.50
		3	First derivative	y = 0.9424x + 0.8368	R² = 0.9850	110.19	3.71	3.57
		3	Second derivative	y = 0.9416x + 0.8863	R² = 0.9863	137.90	4.15	3.41
PEO3 REO3 (PEO*3* adulterated REO3)	PLSR	3	Raw	y = 0.9779x + 0.309	R² = 0.9971	45.63	2.39	1.50
		3	First derivative	y = 0.9516x + 0.6694	R² = 0.9909	87.62	3.30	2.84
		3	Second derivative	y = 0.9593x + 0.5599	R² = 0.9956	55.95	2.64	1.84
	PCR	3	Raw	y = 0.9779x + 0.3086	R² = 0.9971	44.14	2.35	1.50
		3	First derivative	y = 0.9516x + 0.6695	R² = 0.9908	64.04	2.83	2.84
		3	Second derivative	y = 0.9518x + 0.6974	R² = 0.9906	66.83	2.89	2.70
GEO1 REO1 (GEO1 Adulterated REO1)	PLSR	3	Raw	y = 0.9921x + 0.0928	R² = 0.9997	6.00	0.87	0.39
		3	First derivative	y = 0.9778x + 0.3265	R² = 0.9987	15.83	1.40	1.20
		3	Second derivative	y = 0.9708x + 0.4261	R² = 0.9961	47.13	2.43	1.89
	PCR	3	Raw	y = 0.9916x + 0.1038	R² = 0.9997	2.62	0.57	0.41
		3	First derivative	y = 0.9777x + 0.3203	R² = 0.9987	18.51	1.52	1.22
		3	Second derivative	y = 0.9708x + 0.4262	R² = 0.9961	43.05	2.32	1.89
GEO2 REO2 (GEO2 Adulterated REO2)	PLSR	3	Raw	y = 1.0003x − 0.0249	R² = 0.9998	1.49	0.43	0.28
		3	First derivative	y = 0.9799x + 0.2364	R² = 0.9992	10.38	1.14	1.02
		3	Second derivative	y = 0.9783x + 0.1424	R² = 0.9987	15.43	1.39	1.23
	PCR	3	Raw	y = 1.0014x − 0.0348	R² = 0.9998	1.58	0.44	0.29
		3	First derivative	y = 0.9716x + 0.3795	R² = 0.9987	15.06	1.37	1.39
		3	Second derivative	y = 0.972x + 0.4117	R² = 0.9971	27.31	1.85	1.67
GEO3 REO3 (GEO3 Adulterated REO3)	PLSR	3	Raw	y = 1.0067x − 0.2283	R² = 0.9998	2.17	0.52	0.34
		3	First derivative	y = 0.9856x + 0.1796	R² = 0.9997	4.62	0.76	0.67
		3	Second derivative	y = 0.9844x + 0.1644	R² = 0.9991	11.11	1.18	0.82
	PCR	3	Raw	y = 0.9988x − 0.0033	R² = 0.9998	2.33	0.54	0.34
		3	First derivative	y = 0.9857x + 0.179	R² = 0.9997	4.76	0.77	0.67
		3	Second derivative	y = 0.9847x + 0.2223	R² = 0.9989	11.74	1.21	0.82
PEOH REO1 (PEOH Adulterated REO1)	PLSR	3	Raw	y = 0.9806x + 0.4322	R² = 0.9983	6.03	0.74	0.38
		3	First derivative	y = 0.9913x + 0.3094	R² = 0.9996	4.37	0.87	0.39
		3	Second derivative	y = 0.9447x + 1.0213	R² = 0.9881	98.56	3.51	2.87
PEOH REO1 (PEOH Adulterated REO1)	PCR	3	Raw	y = 0.9804x + 0.4348	R² = 0.9981	5.80	0.73	0.39
		3	First derivative	y = 0.9927x + 0.2686	R² = 0.9996	4.26	0.85	0.42
		3	Second derivative	y = 0.9447x + 1.022	R² = 0.9881	115.52	3.80	2.88

## Data Availability

The datasets generated for this study are available on request to the corresponding author.
